# Standardizing phenotypic algorithms for the classification of degenerative rotator cuff tear from electronic health record systems

**DOI:** 10.1093/jamiaopen/ooaf014

**Published:** 2025-03-18

**Authors:** Simone D Herzberg, Nelly-Estefanie Garduno-Rapp, Henry H Ong, Srushti Gangireddy, Anoop S Chandrashekar, Wei-Qi Wei, Lance E LeClere, Wanqing Wen, Katherine E Hartmann, Nitin B Jain, Ayush Giri

**Affiliations:** Vanderbilt University School of Medicine, Nashville, TN 37203, United States; Division of Epidemiology, Department of Medicine, Vanderbilt University Medical Center, Nashville, TN 37203, United States; Clinical Informatics Center, University of Texas Southwestern Medical Center, Dallas, TX 75390, United States; Department of Biomedical Informatics, Vanderbilt University Medical Center, Nashville, TN 37203, United States; Department of Biomedical Informatics, Vanderbilt University Medical Center, Nashville, TN 37203, United States; Vanderbilt University School of Medicine, Nashville, TN 37203, United States; Department of Biomedical Informatics, Vanderbilt University Medical Center, Nashville, TN 37203, United States; Department of Orthopaedic Surgery, Vanderbilt University Medical Center, Nashville, TN 37203, United States; Division of Epidemiology, Department of Medicine, Vanderbilt University Medical Center, Nashville, TN 37203, United States; Department of Biostatistics, Vanderbilt University Medical Center, Nashville, TN 37203, United States; Center for Clinical and Translational Science, University of Kentucky, Lexington, KY 40506, United States; Department of Physical Medicine and Rehabilitation, University of Michigan, Ann Arbor, MI 48109, United States; Division of Epidemiology, Department of Medicine, Vanderbilt University Medical Center, Nashville, TN 37203, United States; Division of Quantitative and Clinical Sciences, Department of Obstetrics and Gynecology, Vanderbilt University Medical Center, Nashville, TN 37203, United States

**Keywords:** rotator cuff, electronic health records, algorithms, shoulder, sports injury

## Abstract

**Objectives:**

Degenerative rotator cuff tears (DCTs) are the leading cause of shoulder pain, affecting 30%-50% of individuals over 50. Current phenotyping strategies for DCT use heterogeneous combinations of procedural and diagnostic codes and are concerning for misclassification. The objective of this study was to create standardized phenotypic algorithms to classify DCT status across electronic health record (EHR) systems.

**Materials and Methods:**

Using a de-identified EHR system, containing chart level data for ∼3.5 million individuals from January 1998 to December 2023, we developed and validated 2 types of algorithms—one requiring and one without imaging verification—to identify DCT cases and controls. The algorithms used combinations of International Classification of Diseases (ICD) / Current Procedural Terminology (CPT) codes and natural language processing (NLP) to increase diagnostic certainty. These hand-crafted algorithms underwent iterative refinement with manual chart review by trained personnel blinded to case-control determinations to compute positive predictive value (PPV) and negative predictive value (NPV).

**Results:**

The algorithm development process resulted in 5 algorithms to identify patients with or without DCT with an overall predictive value of 94.5%: (1) code only cases that required imaging confirmation (PPV = 89%), (2) code only cases that did not require imaging verification (PPV = 92%), (3) NLP-based cases that did not require imaging verification (PPV = 89%), (4) code-based controls that required imaging confirmation (NPV = 90%), and (5) code and NLP-based controls that did not require imaging verification (NPV = 100%). External validation demonstrated 94% sensitivity and 75% specificity for the code-only algorithms.

**Discussion:**

This work highlights the inaccuracy of previous approaches to phenotypic assessment of DCT reliant solely on ICD and CPT codes and demonstrate that integrating temporal and frequency requirements, as well as NLP, substantially increases predictive value. However, while the inclusion of imaging verification enhances diagnostic confidence, it also reduces sample size without necessarily improving predictive value, underscoring the need for a balance between precision and scalability in phenotypic definitions for large-scale genetic and clinical research.

**Conclusions:**

These algorithms represent an improvement over prior DCT phenotyping strategies and can be useful in large-scale EHR studies.

## Introduction

Rotator cuff disease (RCD) or rotator cuff syndrome (RCS) is a composite term often used to describe multiple related pathologies of the rotator cuff, including subacromial pain (impingement) syndrome, rotator cuff tendinopathy, and symptomatic partial and full-thickness rotator cuff tears (RCTs).^1^ It is among the most common causes of pain and disability.^2^ However, the heterogeneity of the term RCD is a major limitation of rotator cuff research as it includes a variety of conditions as a composite outcome, therefore associating risk factors uniformly across all included conditions despite meaningfully different pathophysiologic mechanisms of each injury. Importantly, RCTs (a sub-category of RCD) range in presentation from debilitating pain to asymptomatic incidental findings with some studies reporting rates of asymptomatic tear that account for up to 65% of all tears, making it difficult to assess tear status.^3^^,^^4^

Importantly, current studies investigating RCTs rely either on hospital-based case-control/cohort studies,^5–15^ which are time-consuming and expensive and result in small sample sized, or single diagnostic codes^16–19^ for the classification of RCTs and as a result suffer from high risk of misclassification. Moreover, studies using procedural and diagnostic codes suffer from 3 main limitations: (1) Phenotypic heterogeneity across studies as outlined above. (2) Lack of validated algorithms specifically for DCTs that can be used broadly across institutions utilizing electronic health records (EHRs), so genome-wide association studies to date do not use consistent definitions.^10^^,^^11^^,^^16^^,^^18^ (3) Current phenotyping strategies have involved use of single International Classification of Diseases (ICD)-9/10 and Read v2/v3 codes.^16–19^ Importantly, these codes are recognized to be imprecise (as is quantified later in this paper) and often assigned to patients prior to definitive musculoskeletal diagnoses.^20^ Therefore, reliance on a single occurrence of a diagnostic or procedural code does not provide a reliable phenotypic definition, a problem also highlighted by work from our group.^21^ However, while an important first step in identifying key features from EHRs that are predictive of cuff tear status individually, this previous work does not provide clearly defined algorithms (specific combinations of structured or unstructured codes) that can be applied and tested to classify cases and controls for broader use in EHRs across the United States. To date, only one EHR-based study, conducted by Yanik et al.,^22^ has limited their phenotypic definition to include only procedural codes specific to degenerative cuff repair and employed temporal limitations to ensure exclusion of traumatic tears. However, this study did not perform manual validation of their algorithm. To our knowledge, there is no validated, published algorithm that can be used as standardized methods to classify DCT across different EHR systems. This is particularly important as adoption of EHRs for high-throughput research is on the rise. This work builds on previous work to develop and validate algorithms for application in EHRs allowing varying levels of query. Table 1 summarizes the main phenotypic definition categories currently used in studies evaluating genetic predisposition to RCTs and provides a non-comprehensive list of studies in each category.

**Table 1.** Phenotypic assessment of currently published studies on genetics of rotator cuff tear.

Phenotypic definition	Study example	Phenotype	Sample size
Limited to ICD/CPT codes specific to rotator cuff surgery and excluding traumatic events	Yanik et al., 2021^22^	Degenerative rotator cuff disease	2917 RCD surgery cases and 14 158 matched controls
Single mention of a procedural or nonspecific diagnostic code only	Tashjian et al., 2009^17^	Rotator cuff syndrome (acute or chronic)	3091 cases
	Roos et al., 2017^18^	Rotator cuff injury (acute or chronic)	8357 cases and 94 622 controls
	Tashjain et al., 2021^16^ and Liu et al., 2024^19^	Rotator cuff injury (acute or chronic)^a^	5701 cases and 406 310 controls
Hospital-based cohort study or case control	Harvie et al., 2004^6^ and Gwilym et al., 2009^7^	Full-thickness rotator cuff tear diagnosed on ultrasound	205 cases
	Tashjian et al., 2014^8^	MRI-confirmed, symptomatic, full-thickness rotator cuff tear	92 cases and 92 age-matched controls
	Motta et al., 2014^9^	Rotator cuff disease established by clinical examination and imaging (radiography and MRI)	203 cases and 207 volunteer controls
	Teerlink et al., 2015^10^^a^	Caucasian patients with a MRI confirmed full-thickness rotator cuff tear	175 cases and 3213 controls
	Tashjian et al., 2016^11^^a^	MRI confirmed full-thickness rotator cuff tear	323 cases and 3213 controls
	Assunção et al., 2017^12^	Patients younger than 65 years who underwent repair of full-thickness rotator cuff tears	64 cases and 64 controls
	Kluger et al., 2017^13^	Arthroscopically confirmed large to massive rotator cuff tear chronic	155 cases and 76 controls
	Longo et al., 2018^14^	Caucasian patients undergoing surgery for rotator cuff tears	93 cases and 206 controls
	An et al., 2022^5^	Surgically repaired, full-thickness rotator cuff tear	20 cases and 20 controls

CPT, Current Procedural Terminology; ICD, International Classification of Diseases; MRI, magnetic resonance imaging; RCD, rotator cuff disease.

a Same population.

Digitization of EHRs and linkage with biorepositories, coupled with widespread advances in biomedical informatics now allow for unprecedented access to large-scale healthcare data. Leveraging EHR systems and linked biorepositories along with principled phenotyping approaches can provide larger sample sizes and can allow inquisition of research questions that were previously intractable.^23–25^ As recently demonstrated by our team for diabetic retinopathy, this approach has the potential to advance clinical and etiological understanding of health conditions.^26^ However, to date, there are no validated phenotypic algorithms designed to classify RCT status using EHRs, and current phenotypic selection strategy for RCTs rely on a heterogeneous combination of billing and diagnostic codes,^16–19^^,^^22^ which may identify composite outcome variables for rotator cuff disease rather than tear, leading to concern for misclassification of cases and controls. In addition to heterogeneity due to outcome definition, substantial variability in data availability constraints across EHR systems (availability of notes, access to images, access to billing codes only, etc) further prevent transferability and reproducibility of algorithms. Recognizing these challenges, this study provides principled approaches to conduct high-quality, reproducible research on RCT through the development of validated EHR algorithms for identifying DCT cases and controls.

## Methods

**Figure 1.**
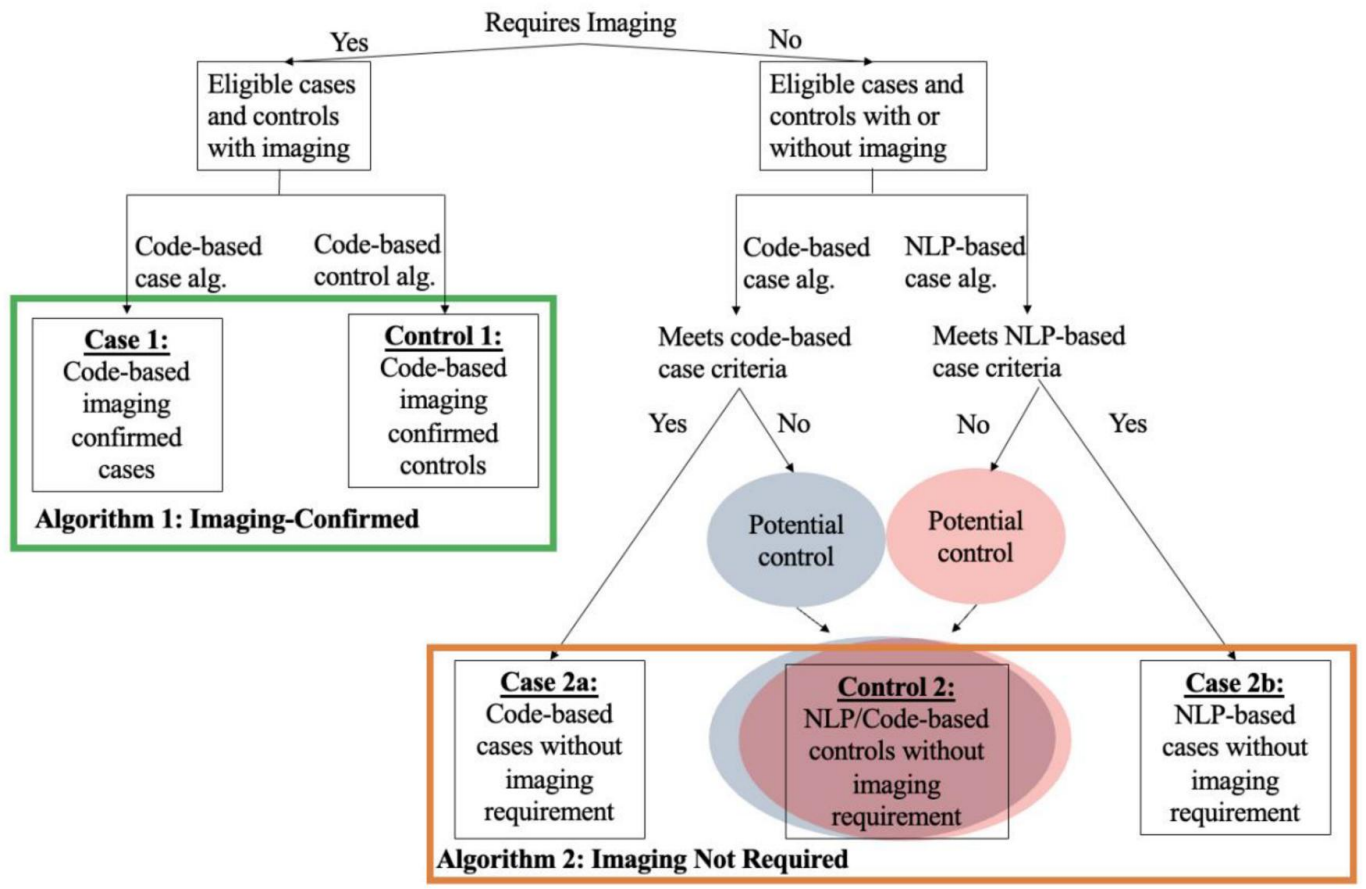
Flow chart for classification of cases and controls by algorithm.

We developed and validated multiple algorithms to identify cases of DCT and controls utilizing the Vanderbilt University Medical Center de-identified EHR database, the Synthetic Derivative (SD) to accommodate applicability to different EHR systems that have varying degrees of constraints in data availability. We used the SD, a de-identified database derived from Vanderbilt’s electronic medical records, containing clinical data for over 3.5 million individuals,^25^ to develop and validate 2 broad types of algorithms that optimize classification of degenerative rotator cuff tear (DCT)—one type requiring evidence of image verification (as documented by presence of tear on magnetic resonance imaging (MRI), computed tomography (CT)-arthrogram, or Ultrasound of the shoulder in EHR record) referred here on out as “imaging-confirmed,” and other that did not require chart documented image verification, referred to as “imaging not required” (Figure 1). Recognizing varying limits in access, and computational capability, we designed the non-imaging-based algorithms with the option of utilizing natural language processing (NLP), consisting of regular-expression based searches for pre-specified phrases, to perform mining of charts, referred here on out as “NLP-based algorithm.”. NLP code was written in python and SQL on Databricks (Azure Cloud environment) and was implemented using regular expression rules. Any algorithms that do not utilize NLP and is entirely reliant on diagnostic and procedural codes such as ICD-9, ICD-10, and Current Procedural Terminology (CPT) codes will be referred to as “code-based” algorithms. Among the algorithms that do not require imaging, we additionally evaluated the impact of incorporating natural language processing (NLP) on sample size and predictive values (Figure 1). All records for Vanderbilt University Medical Center (VUMC) SD participants over the age of 40, from January 1998 up until October 2023 were queried for development of the algorithms.

### Development

**Figure 2.**
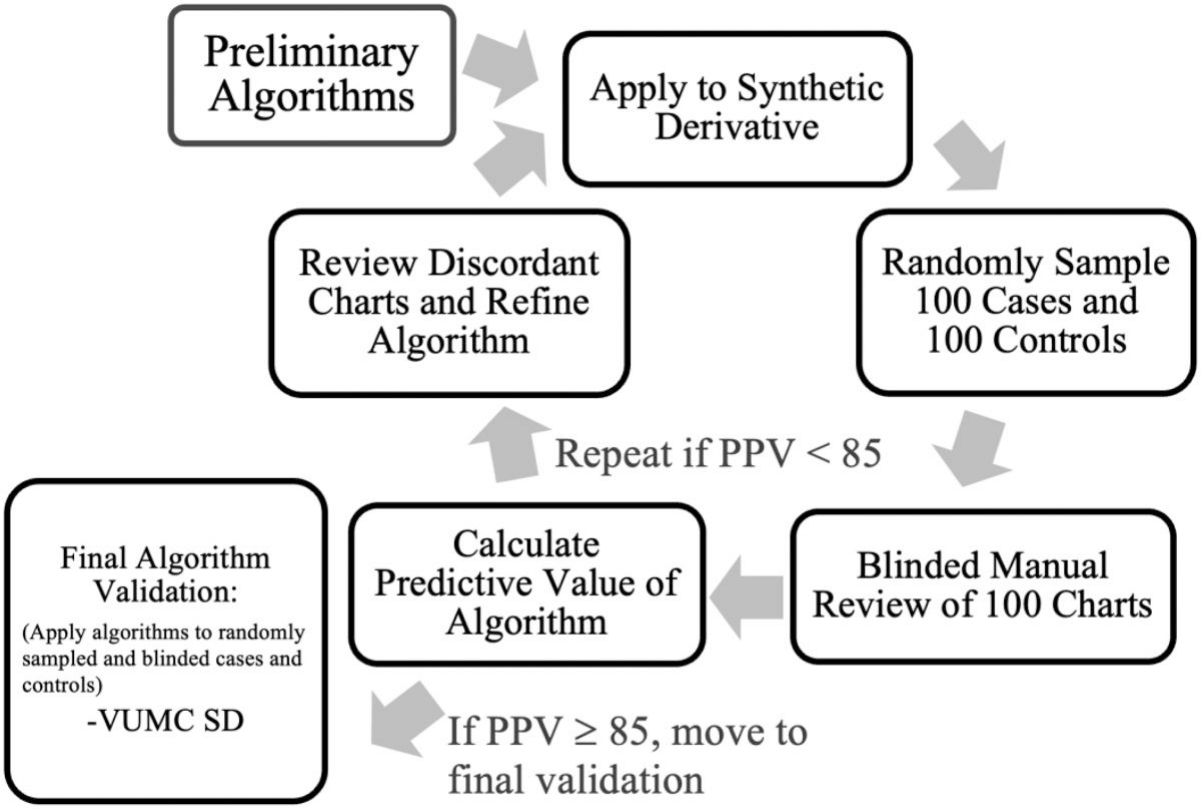
Algorithm development.

The algorithm development process, which took place in the VUMC SD, included application of preliminary algorithms, several rounds of manual review, performance testing, algorithm refining and re-evaluation, and then final validation. A flowchart summarizing the process for algorithm development and validation is shown in Figure 2. This was an iterative development process that resulted in several rounds of assessment and over 4500 case and control charts reviewed before achieving final algorithm status and conducting formal validations.

Informed by previous work,^21^ clinical experience, and a priori knowledge of medical record documentation of rotator cuff diseases, each phenotype consisted of an initial set of sub-criteria designed to capture (for cases) or exclude (for controls) a substantial variety of ways in which RCTs could have been documented in the EHR system. Each of these initial phenotypes underwent 5 full rounds of iterative manual review validation before final formatting for formal blinded dual validation. Each round of review consisted of a random selection of 100 cases and 100 controls from each different case and control phenotype. Over the course of iterative review, a total of 4500 charts were reviewed. Throughout the iterative review, the phenotypes were amended to optimize performance, and any sub-criteria with less than 85% predictive value were either modified and re-validated or dropped from analysis before being formalized for final review. The results of the review process and final phenotype structure are outlined below for each of the 5 phenotypes.

### Validation

Upon satisfactory development of primary algorithms (at least 85% predictive value during iterative review), the resulting final algorithms, presented in the results, were internally validated by applying the algorithm to the entire SD database and then randomly selecting 100 algorithmically determined cases and controls from each sub-criteria. This process resulted in a final validation set of 700 charts, 400 cases (100 NLP-based imaging not required cases, 200 code-based imaging not required cases, and 100 code-based imaging confirmed cases) and 300 controls (100 code-based imaging confirmed controls, and 200 code and NLP-based imaging not required controls). Moreover, 200 code-based image not required cases and 200 code and NLP-based imaging not required controls were selected in order to allow sufficient sizes to assess the predictive value of each sub-criteria. A stratified random sampling strategy was selected to ensure sufficient number of cases and controls were selected from each sub-category to allow for a more precise estimation of predictive values for both cases and controls.

### Manual review criteria

Randomly chosen case and control samples were combined, and manual chart review was performed to determine true case and control status by 2 trained reviewers who were blinded to algorithm-based determinations of case-controls status. Reviewers, who had access to the patient’s de-identified electronic medical record, read through all provider notes, imaging reports, surgical procedural notes, and outside medical record notes/communications, to determine true case control status. Patients were included as a case if they had a diagnosis of a RCT as documented by mention of rotation cuff tear in an imaging report, indication of prior rotator cuff repair or surgical note indicating rotator cuff repair, or a provider documented confirmation of RCT diagnosis. Patient were excluded as cases if the cuff tear was documented as acute, or if there was indication of a major traumatic shoulder event directly preceding the tear diagnosis without indication of antecedent cuff pathology. Patient were included as controls if there was no evidence of a RCT anywhere in their electronic medical record. Patient were included as image-confirmed controls if they had an imaging report that documented an intact rotator cuff, or if they had a provider note that indicated they underwent an imaging or surgical procedure and were noted to have an intact cuff.

After dual review, any discordant charts were resolved by an orthopedic surgeon who served as the expert third-party reviewer. Resultant case versus control assessment was then used to calculate the final Positive Predictive Value (PPV = true positives/[true positives+ false positives]), Negative Predictive Value (NPV= true negatives/[true negatives + false negatives]), as well as the C-statistic (C= true-positive rate/1 − false-positive rate).^27^

### External validation

External validation was conducted using participants from CuffGen study recruited at the University of Texas Southwestern (UTSW). Because of the querying abilities of the UTSW EHR system, only the code-based (imaging-confirmed and imaging not required) algorithms could be applied for validation. Briefly, CuffGen is an ongoing multi-center NIH-funded study recruiting RCT cases and controls across 4 medical centers across the United States: VUMC, UTSW, Massachusetts General Brigham, and University of Michigan. At the time of external validation, the USTW portion of the CuffGen study consisted of 492 participants (405 cases of RCTs and 87 controls). Cases were over the age of 40 and had evidence of cuff tear on MRI that was atraumatic in nature based on chart documented history of disease. Controls consisted of individuals who presented for the evaluation of shoulder pathology that were over the age of 40 with MRI verified lack of RCT. Notably, controls could have had a condition other than a cuff tear, such as adhesive capsulitis, osteoarthritis, or shoulder instability. Because the algorithm was applied to the CuffGen cohort, with a case-control status determined a priori, sensitivity and specificity were used to evaluate the performance of the algorithm.

## Results

### Description of algorithms

We describe 5 distinct algorithms (3 for cases and 2 for controls): (1) code-based, imaging-confirmed cases, (2) code-based imaging not required cases, (3) NLP-based imaging not required cases, (4) code-based imaging-confirmed controls, and (5) code and NLP-based controls that did not require imaging. An overview of the inclusion and exclusion criteria for each of these algorithms is listed in Table 2 and details are described in Tables S1-S5. A full list of the CPT and ICD codes are listed in Table S6.

**Table 2.** Algorithm definitions.

**Code-based, imaging confirmed case sub-criteria**

**Age ≥ 40** **AND** {[CPT code or ICD 9 or ICD 10 code for Shoulder imaging] **AND** [ICD 9 or 10 Code for Diagnosis of Non-Traumatic Rotator Cuff Tear (Within 1 year)]} **AND WITHOUT** {[CPT Code, ICD 9 or ICD 10 code for Traumatic Cuff (PRIOR)] **OR** [CPT code, ICD 9 or 10 code for Non-Tear Related Rotator Cuff Injury (PRIOR)]}

**Code-based, imaging not required case sub-criteria**

**Age ≥ 40** **AND** {[CPT Code for Rotator Cuff Specific Surgery] **OR** [CPT code for Shoulder Surgery **AND** ICD 9 or 10 Code for Diagnosis of Non-Traumatic Rotator Cuff Tear (Within 1 year)] **OR** [Three or more unique visits with mention of ICD 9 or 10 Code for Diagnosis of Non-Traumatic Rotator Cuff Tear]} **AND WITHOUT** [CPT Code for Traumatic Cuff or Non-Rotator Cuff Shoulder Surgery (PRIOR) **OR** ICD 9 or 10 for Traumatic Tear or Non-Tear Related Rotator Cuff Injury (PRIOR)]

**NLP-based, imaging not required case sub-criteria**

**Age ≥ 40** **AND** {[Include if evidence of rotator cuff tear **AND** no evidence of normal rotator cuff after inclusion phraseology] **OR** [Include if evidence of rotator cuff surgery]} **AND WITHOUT** [Evidence of prior acute rotator cuff injury]

**Code-based, imaging confirmed controls sub-criteria**

[Any non-case >40] **AND** {[CPT code for Shoulder imaging] **OR** [ICD 9 or 10 for Shoulder imaging]} **WITHOUT** {[CPT code for Rotator Cuff Surgery] **OR** [ICD 9 for Rotator Cuff Tear] **OR** [ICD10 for Rotator Cuff Tear]}

**Code-based, imaging not required control sub-criteria**

Any non-case from the code- or NLP-based, imaging not required case definitions that is above the age of 40

CPT, Current Procedural Terminology; ICD, International Classification of Diseases; NLP, natural language processing.

### Code*-*based, imaging confirmed case phenotype

The code-based imaging confirmed algorithm consists of one sub-criterion which was defined as the presence of a code (either ICD or CPT) for an imaging modality (MRI, CT-arthrogram, or Ultrasound of the shoulder), followed by a code for RCT within 1 year after the imaging code. This sub-criterion performed very well on the initial review and achieved a PPV of 92%. Because the algorithm was already performing well on initial review, very little modification was made to the imaging confirmed case algorithm during iterative review. The full imaging confirmed code case phenotype is listed in Table S1.

### Code-based, imaging not required, cases

The initial imaging not required code only case phenotype consisted of 8 different sub-criteria (Table S2) designed to comprehensively capture all methods that providers might document RCT status in the EHR system. Possible approaches for the identification of cases in the initial algorithm consisted of (1) surgical confirmation, (2) provider diagnosis, and (3) physical therapy for RCT. The 8 different initial sub-criteria range from CPT codes specific to rotator cuff surgery (the most specific sub-criteria) to a single mention of an ICD code for a RCT (the least specific sub-criteria). These initial algorithms were designed to capture as many cases of RCT as possible, and thus, the predictive value of these algorithms after the first round of manual review varied greatly, from 31% PPV for the single ICD-based sub-criteria to 89% PPV for the RCT specific surgery CPT code-based sub-criteria (Table S2). Moreover, sub-criteria 4a was designed to evaluate the accuracy of the current phenotypic approaches utilized in the field consisting of a single ICD code to assess case status. This approach resulted in the lowest PPV of any sub-criteria at 31%.

After iterative review, 4 of the sub-criteria failed to meet the 85% PPV threshold and were excluded from consideration under final validation. These criteria included both of the sub-criteria using codes for physical therapy (ICD-based or CPT-based) as a diagnostic modality for capturing cases (PPV 42% and 63%, respectively) and the ICD-based definitions that only required 1 or 2 unique visits for RCT only (PPV 31% and 36%, respectively). Therefore, the final code-based imaging not required case algorithm consisted of 4 sub-criteria which are fully elucidated in Table S2 and summarized in Table 2.

### NLP*-*based, imaging not required case phenotypes

The initial imaging not required NLP case phenotype consisted of 15 different inclusion sub-criteria designed to capture the various ways that a medical professional might document the presence of a RCT in the EHR. In addition to these 15 different affirmatory sub-criteria, the NLP algorithm also included 7 different exclusion sub-criteria, designed to capture the various ways that a provider might document either a traumatic RCT or the lack of a RCT in the EHR. Unlike the initial code-based algorithms, which demonstrated a wide range of predictive values, the NLP-based algorithms had a much narrower span, ranging from 80% to 100%.

Only one sub-criterion was completely removed from the NLP-based code definition, the physical examination-based inclusion criterion, because it only captured 15 patients. However, because the remaining NLP-based algorithm performed remarkably well during initial review, it was modified very little from the original algorithm and only slight modifications to remove redundancy, include more specific terms, and allow for plurality or past tense (such as supraspinatus AND teres minor were torn) were made. During this modification process, 2 of the sub-criteria were condensed into 1, ultimately resulting in a final NLP case algorithm of 13 inclusion terms and 7 exclusion terms which are fully listed in Table S3.

### Code*-*based, imaging confirmed control algorithm

Similar to the code-based imaging confirmed case algorithm, the code-based imaging confirmed control algorithm consisted only of one sub-criteria which was defined as any individual over the age of 40 who was not included as a code-based image-confirmed or imaging not required case, who additionally had an ICD or CPT code for imaging but who did NOT have the presence of an ICD code for a RCT at any point after the imaging code. This sub-criterion consistently achieved 100% NPV on initial review and thus did not need to be modified during the iterative review process. The full imaging confirmed code control algorithm is listed in Table S4.

### Code- *±* NLP*-*based, imaging not required control phenotypes

Within the code-based, imaging not required control phenotype, the initial algorithm consisted of 2 different sub-criteria designed to capture the various levels of confidence in control status. The first sub-criteria included any non-case from the code or NLP non-image algorithms that were over the age of 40. The second sub-criteria consisted of individuals over 40 without evidence of a broader list of ICD/CPT codes for RCT (Tables S5 and S6). Both of these sub-criteria performed very well and had 95% and 90% NPV upon initial validation, respectively (Table S5).

Although both sub-criteria of the code-based, imaging not required control definition performed well on iterative review and achieved greater than 85% predictive value, it was found that all controls captured by the second definition were also included in the first control definition. Thus, the second sub-criteria was dropped from the final algorithm because of its redundancy. Therefore, the final code-based imaging not required control algorithm consisted of only one sub-criteria (any participant over the age of 40 who was not identified as a case by either the NLP- or code-based non-image phenotypes).

### Final algorithm performances

The final algorithms underwent blinded review of 700 charts (400 cases and 300 controls). Among the 400 cases, 100 cases were selected from the code image-required algorithm, 100 were chosen from the NLP non-image required algorithm, and 200 cases were selected from the code non-image required algorithm to allow sufficient cases to evaluate predictive values for each sub-criteria of the algorithm. Among the 300 controls, 100 controls were selected from the image-required algorithm and 200 were selected from the non-image required algorithm. Three records (1 case and 2 controls) were excluded because they had no data available in the synthetic derivative and 3 additional records (all classified by the algorithm as controls) were deemed inconclusive because the data available in the chart were not adequate to rule in or out a RCT. There was a 93.1% agreement among the reviewers with only 42 discordant charts (Table 3).

**Table 3.** Final algorithm counts and predictive values in VUMC SD.

	Imaging confirmed		Imaging not required		
	Cases (Code)	Controls (Code)	Cases (Code)	Cases (NLP)	Controls (Code and NLP)
Total count (N)	2632	13 163	8482	54 846	1 756 873
Predictive value % (number correctly classified/total randomly selected from algorithm classification) [95% confidence interval]	89% (89/100) [82.7%-95.1%]	90% (90/100) [84.1%-95.9%]	92% (183/199) [88.2%-95.7%]	89% (89/100) [82.7%-95.1%]	100% (195/195) [100%-100%]

NLP, natural language processing.

Overall, the predictive value of the algorithm that did require imaging was 89.5% (179/200) with a PPV of 89% for code-based imaging confirmed cases and a NPV of 90% for code-based imaging not required controls. For the imaging confirmed cases, the C-statistic was 0.921 (0.846-0.995). The imaging not required algorithm has an overall predictive value of 94.53% (464/494) with a PPV of code-based imaging not required cases of 91.97%, NLP-based imaging not required cases of 89.00%, and a NPV of code- and NLP-based imaging not required controls of 100% (Table 3). For the imaging not required algorithm, the C-statistic was 0.975 (0.946-1.005) and 0.945 (0.887-1.003) for code-based and NLP-based, respectively.

### External validation

When applied to the pre-selected cohort at UTSW, the algorithm identified 420 true positives (TP), 26 false negatives (FN), 11 false positives (FP), and 35 true negatives (TN). This resulted in a sensitivity of 94%, specificity of 76%, and an accuracy of 92%.

## Discussion

Robust phenotypic definitions are of paramount importance in achieving accurate and reliable results, particularly in the context of data derived for research from resources such as EHRs that were not collected for administrative, billing, and clinical assessment. One of the major limitations of existing rotator cuff research in the context of using EHR data for research is the lack of clearly defined algorithms that identify DCT cases and controls. Furthermore, to our knowledge no study has developed, validated, and compared advantages, pitfalls, and consequences of using decision criteria such as using imaging requirement, or NLP in determining the size and composition of the study populations. The development and implementation of the algorithms outlined above represent a significant improvement over prior approaches, which are reliant on a single occurrence of ICD and CPT codes alone. We demonstrated in preliminary evaluations that reliance on single occurrence of ICD codes, as is the current norm in the field, led extremely poor predictive values. In contrast, the addition of temporal and frequency requirements, and NLP in the final algorithms substantially increased predictive value of the algorithms. We were then able to validate the code-based algorithm using a case-control study nested within an external EHR system. Confirmation of this validation for all the approaches described in this paper in additional independent EHR systems will open the door for standardized EHR-based studies of RCTs of unprecedented size. The approaches described here are practical, principled, and overcome limitations of existing approaches, which either lead to poorly defined and misclassified outcomes on one end of the spectrum or give way to time constraints attributed to manual chart review of all records in each study.

One of the key strengths of this study is its versatility of the algorithms, as they offer the choice of using various combinations of structured and unstructured data, making it adaptable across different EHRs. The optional image requirements and ability to include NLP allow for flexibility in application, catering to the diverse data structures and levels of query across various EHRs. This flexibility contributes to its utility in a broad range of clinical contexts, addressing the inherent variability in EHR formats and information availability. While we were able to perform external validation of the code-based algorithm on a limited basis, future work assessing the robustness of all the algorithms presented here in a separate EHR system rather than a in case-control study nested within an EHR system will support the utility and generalizability of these algorithms. Furthermore, the primary objective of this manuscript was to outline the development, describe the criteria, and evaluate the performance of these algorithms validated against chart review. Each algorithm has its advantages and disadvantages, especially in the context of a case-control design and an in-depth discussion related to potential biases for each approach is beyond the scope of this manuscript. Briefly, Table 4 outlines each of the algorithms as well as their advantages and draw backs.

**Table 4.** Algorithm criteria and a priori comparison.

Cases			
	Imaging not required		Imaging confirmed
	Code only	NLP	Code only
**Criteria**	ICD 9/10+CPT codes for rotator cuff tear	Language in the chart noting the presence of rotator cuff tear.	ICD 9/10+CPT codes for imaging followed by codes for rotator cuff tear within one year after imaging code.
**Advantages**	More broadly applicable to EHRs	Larger sample size.	Less misclassification
**Disadvantages**	Higher potential for misclassification	Fewer EHRs have NLP capabilities.	Smaller sample size Validation possible only in EHR-based databases allowing access to reports/notes

Controls		
	Imaging not required	Imaging confirmed
	Code + NLP	Code only
**Criteria**	Lack of ICD 9/10+CPT codes for rotator cuff tear and does not meet either CODE or NLP non-image required case definition.	Lack of ICD 9/10+CPT Codes for rotator cuff tear and ICD/CPT for imaging without code for cuff tear
**Advantages**	Larger sample size; More broadly applicable to EHRs Less risk of selection bias	Less risk of misclassification
**Disadvantages**	Higher risk of misclassification	Limited sample size; Validation possible only in EHR-based databases allowing access to reports/notes Higher risk of selection bias

CPT, Current Procedural Terminology; EHR, electronic health record; ICD, International Classification of Diseases; NLP, natural language processing.

For EHR systems with the capability to include unstructured data, in the form of NLP, the integration of NLP into the non-image required algorithm significantly expanded the sample size of cases without meaningful compromise in predictive value. This augmentation is particularly noteworthy in genetic research as these studies often require extremely large sample sizes to detect modest magnitude of associations between genetic variants and outcome of interest. Large sample sizes enable the identification of these subtle genetic influences, contributing to a more comprehensive understanding of the genetic basis of traits and diseases. Additionally, given that genetic effects may differ among subgroups within a population (eg, different ethnic groups or age categories), larger sample sizes provide the opportunity to investigate these subgroups and identify potential genetic heterogeneity, leading to a more nuanced understanding of genetic influences on traits. With a diverse and large sample, researchers can draw more reliable conclusions about genetic associations that may be generalizable to diverse populations or highlight key differences that exist across genetic ancestries.

Importantly, however, there is often a delicate balance between achieving precision of phenotypic definition and sample size, wherein increasing the stringency of a phenotypic definition often translates to lower case counts. In the case of the algorithms outlined above, since the gold standard for RCT diagnosis is imaging, the image verification component of the algorithm was designed to increase confidence in assessing case status while understanding that this would likely result in decreased sample size. After the final review, as expected, the sample size was significantly limited by the image requirement, however, surprisingly, the requirement for imaging in the algorithm did not result in a higher predictive value than that of the imaging not required algorithms. One potential reason for this difference is the stage of disease captured by each algorithm definition. Cases that have imaging documentation in the EHR were likely in the diagnostic phase of RCT assessment when captured. Because of this, the imaging confirmed case definition captures cases with a degree of uncertainty as to diagnostic status, resulting in more false-positive cases. On the other hand, those captured through imaging not required algorithms have often been diagnosed previously and are being captured either through referral for surgical procedures or from documentation of historical tear/repair. It is impossible for a provider to note all components of a normal medical history/physical examination, and thus, it is very unlikely that an intact RCT would be documented for a patient in the imaging not required group.

## Conclusions

The development and implementation of the algorithms for defining DCT case and control status using EHR data, outlined above, represent a significant improvement over prior strategies, that either rely on single occurrence of ICD or CPT codes, leading to misclassified outcomes, or rely wholly on manual chart review which can be time-consuming. External validation of the code-based algorithm on a limited capacity with a case-control study nested in an EHR system demonstrate promising preliminary results for generalizability of these approaches. A robust and systematic external validation of all of these algorithms in additional EHR systems will inform their utility and generalizability.

## Supplementary Material

ooaf014_Supplementary_Data

## Data Availability

Individual-level from the VUMC SD EHR system are not available due to patient privacy concerns and access restrictions. Access to data may be available to qualified investigators through a collaborative effort for a specified project upon approval from VUMC VICTR.

## Patient involvement

Retrospective electronic health record data was collected for patients seeking care at VUMC who agreed to use of their EHR data for research purposes and signed informed consent. There were no additional interventions or treatments offered to patients in this study.
