# Supplementary material for: Standardizing phenotypic algorithms for the classification of degenerative rotator cuff tear from electronic health record systems
Source: JAMIA Open. 2025 Mar 18;8(2):ooaf014. doi: 10.1093/jamiaopen/ooaf014 (PMC11917214; doi:10.1093/jamiaopen/ooaf014)
Supplement: ooaf014_Supplementary_Data [file ooaf014_supplementary_data.docx]

**Supplemental Figures/Tables:**

| Supplemental Table 1. Code Image-Based Case Sub-Criteria | | | |
| --- | --- | --- | --- |
| Sub-Criteria Definition | | Initial Validation  Predictive Value | |
| 1 | **CPT code for Shoulder imaging**  **OR**  **ICD 9 or 10 for Shoulder imaging**  **and**  **ICD 9 or 10 Code for Diagnosis of Non-Traumatic Rotator Cuff Tear (Within one year)**  **without**  **CPT Code for Traumatic Cuff or Non-Rotator Cuff Shoulder Surgery (PRIOR)**  **without**  **ICD 9 or 10 for Traumatic Tear or Non-Tear Related Rotator Cuff Injury (PRIOR)** | | **92%** |
| This version of the algorithm underwent several iterative validations and the predictive values listed here represent the highest predictive value achieved by this sub-criterion before final validation. This sub-criterion is listed in bold because it achieved greater than the 85% predictive value threshold and was thus included in the final algorithm. | | | |

| Supplemental Table 2. Code Based Non-Image-Based Case Sub-Criteria | | | |
| --- | --- | --- | --- |
| Sub-Criteria Definition | | **Initial Validation**  **Predictive Value** | |
| 1 | **CPT Code for Rotator Cuff Specific Surgery**  **without**  **CPT Code for Traumatic Cuff or Non-Rotator Cuff Shoulder Surgery (PRIOR)**  **without**  **ICD 9 or 10 for Traumatic Tear or Non-Tear Related Rotator Cuff Injury (PRIOR)** | | **95%** |
| 2 | **CPT code for Shoulder Surgery**  **and**  **ICD 9 or 10 Code for Diagnosis of Non-Traumatic Rotator Cuff Tear (Within one year)**  **without**  **CPT Code for Traumatic Cuff or Non-Rotator Cuff Shoulder Surgery (PRIOR)**  **without**  **ICD 9 or 10 for Traumatic Tear or Non-Tear Related Rotator Cuff Injury (PRIOR)** | | **90%** |
| 3a | ICD 9 or 10 for Physical Therapy  and  ICD 9 or 10 Code for Diagnosis of Non-Traumatic Rotator Cuff Tear (Within one year)  without  CPT Code for Traumatic Cuff or Non-Rotator Cuff Shoulder Surgery (PRIOR)  without  ICD 9 or 10 for Traumatic Tear or Non-Tear Related Rotator Cuff Injury (PRIOR) | | 42% |
| 3b | CPT code for Physical Therapy  and  ICD 9 or 10 Code for Diagnosis of Non-Traumatic Rotator Cuff Tear (Within one year)  without  CPT Code for Traumatic Cuff or Non-Rotator Cuff Shoulder Surgery (PRIOR)  without  ICD 9 or 10 for Traumatic Tear or Non-Tear Related Rotator Cuff Injury (PRIOR) | | 63% |
| 4a | One unique visit with mention of ICD 9 or 10 Code for Diagnosis of Non-Traumatic Rotator Cuff Tear (Within one year)  without  CPT Code for Traumatic Cuff or Non-Rotator Cuff Shoulder Surgery (PRIOR)  without  ICD 9 or 10 for Traumatic Tear or Non-Tear Related Rotator Cuff Injury (PRIOR) | | 31% |
| 4b | Two unique visits with mention of ICD 9 or 10 Code for Diagnosis of Non-Traumatic Rotator Cuff Tear (Within one year)  without  CPT Code for Traumatic Cuff or Non-Rotator Cuff Shoulder Surgery (PRIOR)  without  ICD 9 or 10 for Traumatic Tear or Non-Tear Related Rotator Cuff Injury (PRIOR) | | 36% |
| 4c | **Three unique visits with mention of ICD 9 or 10 Code for Diagnosis of Non-Traumatic Rotator Cuff Tear (Within one year)**  **without**  **CPT Code for Traumatic Cuff or Non-Rotator Cuff Shoulder Surgery (PRIOR)**  **without**  **ICD 9 or 10 for Traumatic Tear or Non-Tear Related Rotator Cuff Injury (PRIOR)** | | **87%** |
| 4d | **Four or more unique visits with mention of ICD 9 or 10 Code for Diagnosis of Non-Traumatic Rotator Cuff Tear (Within one year)**  **without**  **CPT Code for Traumatic Cuff or Non-Rotator Cuff Shoulder Surgery (PRIOR)**  **without**  **ICD 9 or 10 for Traumatic Tear or Non-Tear Related Rotator Cuff Injury (PRIOR)** | | **92%** |
| This version of the algorithm underwent several iterative validations and the predictive values listed here represent the highest predictive value achieved by each sub-criteria before final validation. The sub-criteria listed in in bold are those that met the initial predictive value threshold of 85% and were included in the final algorithm. | | | |

| Supplemental Table 3. NLP Based Image Not Required Case Definition | | | |
| --- | --- | --- | --- |
| Sub-Criteria Definition | | **Initial Validation**  **Predictive Values** | |
| Exclusion Criteria* | | | |
| 1 | **Rotator cuff (is)… normal** | | |
| 2 | **Normal rotator cuff** | | |
| 3 | **No (evidence of/discernable/obvious/significant/ partial/ full-thickness/) rotator cuff tear/avulsion/pathology/abnormality(s)** | | |
| 4 | **Rotator cuff … intact** | | |
| 5 | **Traumatic tear of (right/left) rotator cuff** | | |
| 6 | **No tear/torn (of) rotator cuff** | | |
| 7 | **Rotator cuff… no tear** | | |
| Inclusion Criteria | | | |
| 1 | **(complete/partial/full/entire/incomplete/mild/massive/bilateral/small) (thickness) rotator cuff/cuff/supraspinatus (and)/infraspinatus (and)/subscapularis (and)/teres minor(and) (partial/partially/completely/complete) (tendon (s)) (is/has been/has a)(partial/partially/full/fully/complete/completely) (thickness) torn/tear/avulsion/avulsed** | | **100%** |
| 2 | **(complete/partial/full/entire/incomplete/mild/massive/bilateral/small) (thickness) tear of the (tendon(s) of the) rotator cuff/ cuff/Supraspinatus (and)/infraspinatus (and)/subscapularis (and)/teres minor (and)** | | **90%** |
| 3 | **(completely/massively/entirely/fully/ partially) torn rotator cuff/cuff/supraspinatus (and)/infraspinatus (and)/subscapularis (and)/teres minor (and)** | | **90%** |
| 4 | **(complete/massive/entire/full/partial) defect of (the) rotator cuff/cuff/supraspinatus (and) (and)/infraspinatus(and)/subscapularis (and)/teres minor (and)** | | **100%** |
| 5 | **(complete/massive/entire/full/ partial) rotator cuff/cuff/supraspinatus(and)/infraspinatus(and)/subscapularis(and)/teres minor(and) defect(s)** | | **100%** |
| 6** | (full/ partial) rotator cuff/cuff/supraspinatus/infraspinatus/subscapularis/teres minor (tear) repair | | 100% |
| 7 | **rotator cuff/cuff/supraspinatus (and)/infraspinatus (and)/subscapularis (and)/teres minor (and)repair(ed) (arthroscopically)** | | **100%** |
| 8 | **(arthroscopically) repair(ed) (of)(the) rotator cuff/cuff/supraspinatus (and)/infraspinatus (and)/subscapularis (and)/teres minor (and)** | | **100%** |
| 9 | **arthroplasty of rotator cuff/cuff/supraspinatus (and)/infraspinatus (and)/subscapularis (and)/teres minor (and)** | | **100%** |
| 10 | **rotator cuff/cuff/supraspinatus (and)/infraspinatus (and)/subscapularis (and)/teres minor (and) arthroplasty** | | **100%** |
| 11** | **(surgery) (of the) arthroplastic/arthroplasty/arthroscopic (surgery) (of the)? rotator cuff/cuff/supraspinatus (and)/infraspinatus (and)/subscapularis (and)/teres minor (and) ... repair(ed)** | | **100%** |
| 12 | **rotator cuff/cuff/supraspinatus (and)/infraspinatus (and)/subscapularis (and)/teres minor (and) surgery** | | **100%** |
| 13 | **(surgical/surgically) (repair(ed)) (of) (the) rotator cuff/cuff/supraspinatus (and)/infraspinatus (and)/subscapularis (and)/teres minor(and) tear (arthroscopic/arthroplastic) repair/arthroplasty** | | **100%** |
| 14 | **shoulder arthroplasty with repair of (the) rotator cuff/cuff/supraspinatus (and)/infraspinatus (and)/subscapularis (and)/teres minor (and) –** | | **100%** |
| 15 | Physical examination:… Rotator cuff/Empty Can/ Neer/Hawkins/ Lift off/ Belly Press/ Drop Arm/ Bear Hug/ … positive/+ | | 80% |
| This version of the algorithm underwent several iterative validations and the predictive values listed here represent the highest predictive value achieved by each sub-criteria before final validation. The sub-criteria listed in bold met the initial predictive value threshold of 85% and were included in the final algorithm.  () = optional terms  / = interchangeable terms  … = allow for space between terms – 20 characters for exclusion criteria and 55 characters for inclusion criteria  *Exclusion terms were applied to all sub-criteria before inclusion criteria  ** Sub-criterion 6 was removed because it was captured entirely by sub criterion 11 | | | |

| Supplemental Table 4. Code Based Image Confirmed Control Sub-Criteria | | | |
| --- | --- | --- | --- |
| Sub-Criteria Definition | | Initial Validation  Predictive Value | |
| 3 | **Any non-case >40**  **With**  **CPT code for Shoulder imaging**  **OR**  **ICD 9 or 10 for Shoulder imaging**  **Without**  **CPT:**   1. **CPT code 23412: repair of ruptured musculotendinous cuff (eg. Rotator cuff) open; chronic** 2. **OR CPT code 23420: reconstruction of complete shoulder (rotator) cuff avulsion; chronic** 3. **CPT code 23410: repair of ruptured musculotendinous cuff (eg. Rotator cuff) open; acute** 4. **CPT code 29827 and 29828: Arthroscopy shoulder, surgical; with rotator cuff repair**   **ICD 9**   1. **727.61 Complete rupture of rotator cuff** 2. **726.13 Partial tear of rotator cuff** 3. **726.1 Rotator cuff syndrome of shoulder and allied disorders**   **ICD10**   1. **M75.120 Complete rotator cuff tear or rupture of unspecified shoulder, not specified as traumatic** 2. **M75.121 Complete rotator cuff tear or rupture of right shoulder, not specified as traumatic** 3. **M75.122 Complete rotator cuff tear or rupture of left shoulder, not specified as traumatic** 4. **M75.110 Incomplete rotator cuff tear or rupture of unpsecified shoulder, not specified as traumatic** 5. **M75.111 Incomplete rotator cuff tear or rupture of right shoulder, not specified as traumatic** 6. **M75.112 Incomplete rotator cuff tear or rupture of left shoulder, not specified as traumatic** 7. **M75.100 Unspecified rotator cuff tear or rupture of unspecified shoulder, not specified as traumatic** 8. **M75.101 Unspecified rotator cuff tear or rupture of right shoulder, not specified as traumatic**   **M75.102 Unspecified rotator cuff tear or rupture of left shoulder, not specified as traumatic** | | **100%** |
| This version of the algorithm underwent several iterative validations and the predictive values listed here represent the highest predictive value achieved by this sub-criterion before final validation. This sub-criterion is bolded because it met the 85% predictive value threshold and was included in the final algorithm. | | | |

| Supplemental Table 5. Code Based Image Not Required Control Sub-Criteria | | | |
| --- | --- | --- | --- |
| Sub-Criteria Definition | | Initial Validation  Predictive Value | |
| 1 | **Any non-case from the Code or NLP non image required case definitions that is above the age of 40** | | **95%** |
| 2 | Anyone above the age of 40,  without:  CPT:   1. CPT code 23412: repair of ruptured musculotendinous cuff (eg. Rotator cuff) open; chronic 2. OR CPT code 23420: reconstruction of complete shoulder (rotator) cuff avulsion; chronic 3. CPT code 23410: repair of ruptured musculotendinous cuff (eg. Rotator cuff) open; acute 4. CPT code 29827 and 29828: Arthroscopy shoulder, surgical; with rotator cuff repair   ICD 9   1. 727.61 Complete rupture of rotator cuff 2. 726.13 Partial tear of rotator cuff 3. 726.1 Rotator cuff syndrome of shoulder and allied disorders   ICD10   1. M75.120 Complete rotator cuff tear or rupture of unspecified shoulder, not specified as traumatic 2. M75.121 Complete rotator cuff tear or rupture of right shoulder, not specified as traumatic 3. M75.122 Complete rotator cuff tear or rupture of left shoulder, not specified as traumatic 4. M75.110 Incomplete rotator cuff tear or rupture of unpsecified shoulder, not specified as traumatic 5. M75.111 Incomplete rotator cuff tear or rupture of right shoulder, not specified as traumatic 6. M75.112 Incomplete rotator cuff tear or rupture of left shoulder, not specified as traumatic 7. M75.100 Unspecified rotator cuff tear or rupture of unspecified shoulder, not specified as traumatic 8. M75.101 Unspecified rotator cuff tear or rupture of right shoulder, not specified as traumatic 9. M75.102 Unspecified rotator cuff tear or rupture of left shoulder, not specified as traumatic | | 90% |
| This version of the algorithm underwent several iterative validations and the predictive values listed here represent the highest predictive value achieved by these sub-criteria before final validation. Both sub-criterion achieved greater than the 85% predictive value threshold, but the first criterion was the only one included in the final algorithm because it was found to capture everyone listed in the second criterion. | | | |

| **Supplemental Table 6. Full List of ICD and CPT Codes Used in Code Based Algorithms** | | |
| --- | --- | --- |
| Code Type | Code | Code Explanation |
| **Codes for Rotator Cuff Tear** | | |
| CPT | 23412 | repair of ruptured musculotendinous cuff (eg. Rotator cuff) open; chronic |
| CPT | 23420 | reconstruction of complete shoulder (rotator) cuff avulsion; chronic |
| CPT | 23397 | Under Repair, Revision, and/or Reconstruction Procedures on the Shoulder |
| CPT | 29826 | Arthroscopy, shoulder, surgical; decompression of subacromial space with partial |
| CPT | 29901 | Under Endoscopy/Arthroscopy Procedures on the Musculoskeletal System |
| CPT | 29827 | Arthroscopy shoulder, surgical; with rotator cuff repair |
| CPT | 29828 | Arthroscopy shoulder, surgical; with rotator cuff repair |
| CPT | 29822 | Arthroscopy, shoulder, surgical; debridement, limited includes debridement of soft or hard tissue |
| ICD9CM | 727.61 | Complete rupture of rotator cuff |
| ICD9CM | 726.13 | Partial tear of rotator cuff |
| ICD9CM | 83.63 | Rotator cuff repair |
| ICD10CM | M75.120 | Complete rotator cuff tear or rupture of unspecified shoulder, not specified as traumatic |
| ICD10CM | M75.121 | Complete rotator cuff tear or rupture of right shoulder, not specified as traumatic |
| ICD10CM | M75.122 | Complete rotator cuff tear or rupture of left shoulder, not specified as traumatic |
| ICD10CM | M75.110 | Incomplete rotator cuff tear or rupture of unspecified shoulder, not specified as traumatic |
| ICD10CM | M75.111 | Incomplete rotator cuff tear or rupture of right shoulder, not specified as traumatic |
| ICD10CM | M75.112 | Incomplete rotator cuff tear or rupture of left shoulder, not specified as traumatic |
| ICD10CM | M75.100 | Unspecified rotator cuff tear or rupture of unspecified shoulder, not specified as traumatic |
| ICD10CM | M75.101 | Unspecified rotator cuff tear or rupture of right shoulder, not specified as traumatic |
| ICD10CM | M75.102 | Unspecified rotator cuff tear or rupture of left shoulder, not specified as traumatic |
| **Codes for Rotator Cuff Exclusions (Traumatic Tear)** | | |
| CPT | 23410 | repair of ruptured musculotendinous cuff (eg. Rotator cuff) open; acute |
| CPT | 24341 | repair, tendon or muscle, upper arm or elbow, each tendon or muscle, primary or secondary (excludes Rotator cuff) |
| ICD9CM | 840.3 | Infraspinatus (muscle) (tendon) sprain |
| ICD9CM | 840.4 | Rotator cuff (capsule) sprain |
| ICD9CM | 840.5 | Subscapularis (muscle) sprain |
| ICD9CM | 840.6 | Supraspinatus (muscle) (tendon) sprain |
| ICD10CM | S46.011A | Strain of muscle(s) and tendon(s) of the rotator cuff of right shoulder, initial encounter |
| ICD10CM | S46.011D | Strain of muscle(s) and tendon(s) of the rotator cuff of right shoulder, subsequent encounter |
| ICD10CM | S46.011S | Strain of muscle(s) and tendon(s) of the rotator cuff of right shoulder, sequela |
| ICD10CM | S46.012A | Strain of muscle(s) and tendon(s) of the rotator cuff of left shoulder, initial encounter |
| ICD10CM | S46.012D | Strain of muscle(s) and tendon(s) of the rotator cuff of left shoulder, sequential encounter |
| ICD10CM | S46.012S | Strain of muscle(s) and tendon(s) of the rotator cuff of left shoulder, sequela |
| ICD10CM | S46.011A | Strain of muscle(s) and tendon(s) of the rotator cuff of unspecified shoulder, initial encounter |
| ICD10CM | S46.011D | Strain of muscle(s) and tendon(s) of the rotator cuff of unspecified shoulder, subsequent encounter |
| ICD10CM | S46.011S | Strain of muscle(s) and tendon(s) of the rotator cuff of unspecified shoulder, sequela |
| ICD10CM | S46.021A | Laceration of muscle(s) and tendon(s) of the rotator cuff of the right shoulder, initial encounter |
| ICD10CM | S46.021D | Laceration of muscle(s) and tendon(s) of the rotator cuff of the right shoulder, Sequential encounter |
| ICD10CM | S46.021S | Laceration of muscle(s) and tendon(s) of the rotator cuff of the right shoulder, sequela |
| ICD10CM | S46.022A | Laceration of muscle(s) and tendon(s) of the rotator cuff of the left shoulder, initial encounter |
| ICD10CM | S46.022D | Laceration of muscle(s) and tendon(s) of the rotator cuff of the left shoulder, Sequential encounter |
| ICD10CM | S46.022S | Laceration of muscle(s) and tendon(s) of the rotator cuff of the left shoulder, sequela |
| ICD10CM | S46.029A | Laceration of muscle(s) and tendon(s) of the rotator cuff of unspecified shoulder, initial encounter |
| ICD10CM | S46.029D | Laceration of muscle(s) and tendon(s) of the rotator cuff of unspecified shoulder, Sequential encounter |
| ICD10CM | S46.029S | Laceration of muscle(s) and tendon(s) of the rotator cuff of unspecified shoulder, sequela |
| ICD10CM | S43.421A | Sprain of muscle(s) and tendon(s) of the rotator cuff of right shoulder, initial encounter |
| ICD10CM | S43.421D | Sprain of muscle(s) and tendon(s) of the rotator cuff of right shoulder, sequential encounter |
| ICD10CM | S43.421S | Sprain of muscle(s) and tendon(s) of the rotator cuff of right shoulder, sequela |
| ICD10CM | S43.422A | Sprain of muscle(s) and tendon(s) of the rotator cuff of left shoulder, initial encounter |
| ICD10CM | S43.422D | Sprain of muscle(s) and tendon(s) of the rotator cuff of left shoulder, sequential encounter |
| ICD10CM | S43.422S | Sprain of muscle(s) and tendon(s) of the rotator cuff of left shoulder, sequela |
| ICD10CM | S43.429A | Sprain of muscle(s) and tendon(s) of the rotator cuff of unspecified shoulder, initial encounter |
| ICD10CM | S43.429D | Sprain of muscle(s) and tendon(s) of the rotator cuff of unspecified shoulder, sequential encounter |
| ICD10CM | S43.429S | Sprain of muscle(s) and tendon(s) of the rotator cuff of unspecified shoulder, sequela |
| ICD10CM | M12.511 | Traumatic Arthropathy, right shoulder |
| ICD10CM | M12.512 | Traumatic Arthropathy, left shoulder |
| ICD10CM | M12.519 | Traumatic Arthropathy, unspecified shoulder |
| ICD10CM | M75.30 | Calcific Tendinitis of unspecified shoulder |
| ICD10CM | M75.31 | Calcific Tendinitis of right shoulder |
| ICD10CM | M75.32 | Calcific Tendinitis of left shoulder |
| **Codes for Shoulder Imaging** | | |
| CPT | 76880 | [Expired] Ultrasound, extremity, nonvascular, real time with image documentation |
| CPT | 78661 | Ultrasound, complete joint (ie. Joint space and peri-articular soft tissue structures) real time with image documentation |
| CPT | 78662 | Ultrasound, limited, joint or other non-vascular extremity structure (i.e. joint space, peri-articular tendon[s], muscle[s], nerve[s], other soft tissue structure[s], or soft tissue mass[es]) real time with image documentation |
| ICD9CM | 88.94 | Magnetic Resonance Imaging of Musculoskeletal |
| ICD9CM | 88.32 | Contrast arthrogram |
| ICD9CM | 88.7 | Diagnostic Ultrasound |
| ICD10CM | BP3EZZZ | MRI upper extremity left |
| ICD10CM | BP3FYZZ | MRI upper extremity left, with contrast |
| ICD10CM | BP3FY0Z | MRI upper extremity left, enhanced or unenhanced |
| ICD10CM | BP38ZZZ | MRI upper extremity right |
| ICD10CM | BP38YZZ | MRI upper extremity right, with contrast |
| ICD10CM | BP38Y0Z | MRI upper extremity right, enhanced or unenhanced |
| ICD10CM | BL33YZZZ | MRI upper extremity tendon |
| ICD10CM | BL33YZZ | MRI upper extremity tendon with contrast |
| ICD10CM | BL33Y0Z | MRI upper extremity tendon with contrast, enhanced or unenhanced |
| ICD10CM | BL30ZZZ | MRI upper extremity Connective tissue |
| ICD10CM | BL30YZZ | MRI upper extremity Connective tissue with contrast |
| ICD10CM | BL30Y0Z | MRI upper extremity Connective Tissue with contrast, enhanced or unenhanced |
| ICD10CM | B53NZZZ | MRI upper extremity vein |
| ICD10CM | B53NYZZ | MRI upper extremity vein with contrast |
| ICD10CM | B53NY0Z | MRI upper extremity vein with contrast enhanced or unenhanced |
| ICD10CM | B33KZZZ | MRI upper extremity artery |
| ICD10CM | B33KYZZ | MRI upper extremity artery with contrast |
| ICD10CM | B33KY0Z | MRI upper extremity artery with contrast enhanced or unenhanced |
| ICD10CM | B33JZZZ | MRI Left upper extremity artery |
| ICD10CM | B33JYZZ | MRI Left upper extremity artery with contrast |
| ICD10CM | B33JY0Z | MRI Left upper extremity artery with contrast enhanced or unenhanced |
| ICD10CM | B33HZZZ | MRI Right upper extremity artery |
| ICD10CM | B33HYZZ | MRI Right upper extremity artery with contrast |
| ICD10CM | B33HY0Z | MRI Right upper extremity artery with contrast enhanced or unenhanced |
| **Codes for Non-Traumatic Tendon Injury** | | |
| ICD9CM | 727.6 | Rupture of tendon nontraumatic |
| ICD9CM | 727.60 | Nontraumatic rupture of unspecified tendon |
| **Codes for Physical Therapy** | | |
| CPT | 90071 | [Expired] Physical therapy evaluation |
| CPT | 90072 | [Expired] Physical therapy re-evaluation |
| CPT | 97161 | Physical therapy, low complexity, typical time with patient and/or/family 10 mins |
| CPT | 97162 | Physical therapy, moderate complexity, typical time with patient and/or/family 30 mins |
| CPT | 97163 | Physical therapy, high complexity, typical time with patient and/or/family 45 mins |
| CPT | 97164 | Reevaluation of physical therapy established plan of care, requiring these components: An examination including a review of history and use of standardized tests and measures is required; and revised plan of care using a standardized patient assessment instrument or measurable assessment of functional outcome Typically 20 mins are spent with the patient or family. |
| ICD9CM | 93.0 | physical therapy, respiratory therapy, rehabilitation, and related procedures |
| ICD9CM | 93.00 | diagnostic physical therapy |
| ICD9CM | 93.09 | other diagnostic physical therapy procedure |
| ICD9CM | 93.1 | physical therapy procedure |
| ICD9CM | 93.2 | other physical therapy musculoskeletal procedures |
| ICD9CM | 93.38 | combined physical therapy without mention of components |
| ICD9CM | 93.39 | other physical therapy |
| ICD9CM | V57.1 | care involving other physical therapy |
